# Case series: CT scan in cardiac arrest and imminent cardiogenic shock

**DOI:** 10.4103/0971-3026.63037

**Published:** 2010-05

**Authors:** Manisha Jana, Shivanand Ramachandra Gamanagatti, Atin Kumar

**Affiliations:** Department of Radiodiagnosis, All India Institute of Medical Sciences, Ansari Nagar, New Delhi, India

**Keywords:** Cardiac arrest, cardiogenic shock, computed tomography, contrast layering

## Abstract

Imaging a patient having a cardiac arrest on the examination table is not a common occurrence. Altered hemodynamics resulting from pump failure causes stasis of blood in the dependent organs of the body, which is manifested on imaging by dependent contrast pooling and layering. Often a patient with imminent cardiogenic shock also shows a similar dependent contrast pooling and layering, which is a marker of the worsening clinical condition. We report the contrast-enhanced CT scan features in four cases, two of whom had cardiac arrest during imaging, while the other two developed cardiogenic shock soon after the examination. Dependent contrast pooling and layering were found in all of them, with faint or no opacification of the left cardiac chambers. Contrast pooling was noted in the dependent lumbar veins, hepatic veins, hepatic parenchyma, and the right renal vein, as well as in the dependent part of the IVC and the right heart chambers.

## Introduction

Cardiogenic shock is defined as a state of tissue hypoperfusion due to decreased systemic cardiac output as a result of pump failure. Cardiac arrest results in abrupt cessation of cardiac pump function, which may be reversible by prompt intervention but leads to death otherwise. The real incidence of cardiac arrest during the performance of CT scans is unknown, but it is certainly not a very common occurrence. The imaging findings of cardiac arrest during CT scans have been described in only a few case reports.[1–3] Contrast layering in the inferior vena cava (IVC) as a marker of imminent cardiogenic shock has been reported in one case report in the English literature.[4] We describe the imaging features of two cases of cardiac arrest during CT scan and two cases of imminent cardiogenic shock, who showed contrast layering in the IVC on CT scan.

## Case Reports

### Case 1

A 25-year-old man presented to the emergency department with multiple injuries sustained in a road traffic accident. On primary survey, he was hemodynamically unstable and had reduced bilateral air entry on chest auscultation and increasing respiratory distress. He was intubated and connected to a ventilator. Bilateral intercostal tube drainage was also performed. He was hypotensive (blood pressure 90/50 mm Hg). Resuscitation attempts were carried out using large amounts of intravenous (IV) fluids and, under continuous monitoring of the blood pressure and the respiratory rate, a contrast-enhanced CT (CECT) scan of the thorax and abdomen was performed on a 40-channel multislice CT scanner (SOMATOM Sensation, Siemens, Erlangen, Germany) after intravenous injection of 100 ml of non-ionic iodinated contrast material.

**Figure 1(A-D):**
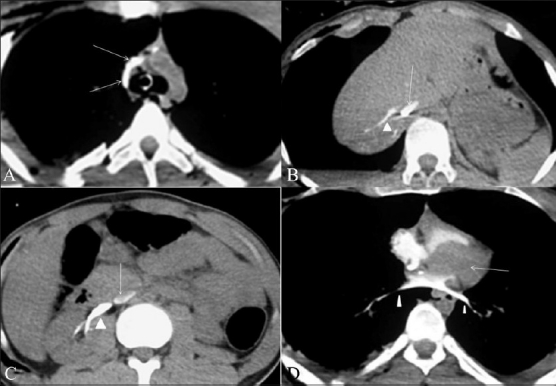
Cardiac arrest in a 25-year-old male with polytrauma. Axial contrast-enhanced CT scans show intense opacification of the SVC and azygos vein (arrows in A). Contrast is seen pooling in the dependent part of the IVC (arrows in B and C), with opacification of the hepatic veins (arrowheads in b) and the right renal vein (arrowhead in C). There is no contrast in the left heart chamber (arrow in D). Contrast opacification of the pulmonary veins is seen (arrowheads in D). Note that there is no parenchymal enhancement of the liver or kidney and no contrast is seen in the aorta (B,C)

The patient had a cardiac arrest during the CT scan. The CT scan showed contrast pooling in the dependent portions of the superior vena cava (SVC) and the inferior vena cava (IVC), the hepatic veins, and the right renal vein [Figure 1A–C], with the formation of a blood-contrast level. The right chambers of the heart were opacified and there was also opacification of the pulmonary veins [Figure 1D]. There was no contrast in the left heart chambers.

The patient had a left iliac wing fracture and multiple rib fractures, with extensive subcutaneous emphysema involving the thoracic wall.

### Case 2

A 26-year-old male came to the trauma center with multiple injuries after a road traffic accident. On primary survey, the airway was patent but the patient had poor respiratory effort and was hemodynamically unstable. He was intubated and put on artificial respiration and IV fluids were transfused. He continued to remain hemodynamically unstable (BP: 80/50 mm Hg). He had multiple bony injuries involving both lower limbs and the left upper limb. CECT scan of the thorax and abdomen was performed after injecting 80 ml of non-ionic iodinated contrast material under continuous monitoring of BP and respiration. He had a cardiac arrest during the CT scan and, despite resuscitative attempts, died.

**Figure 2(A-G):**
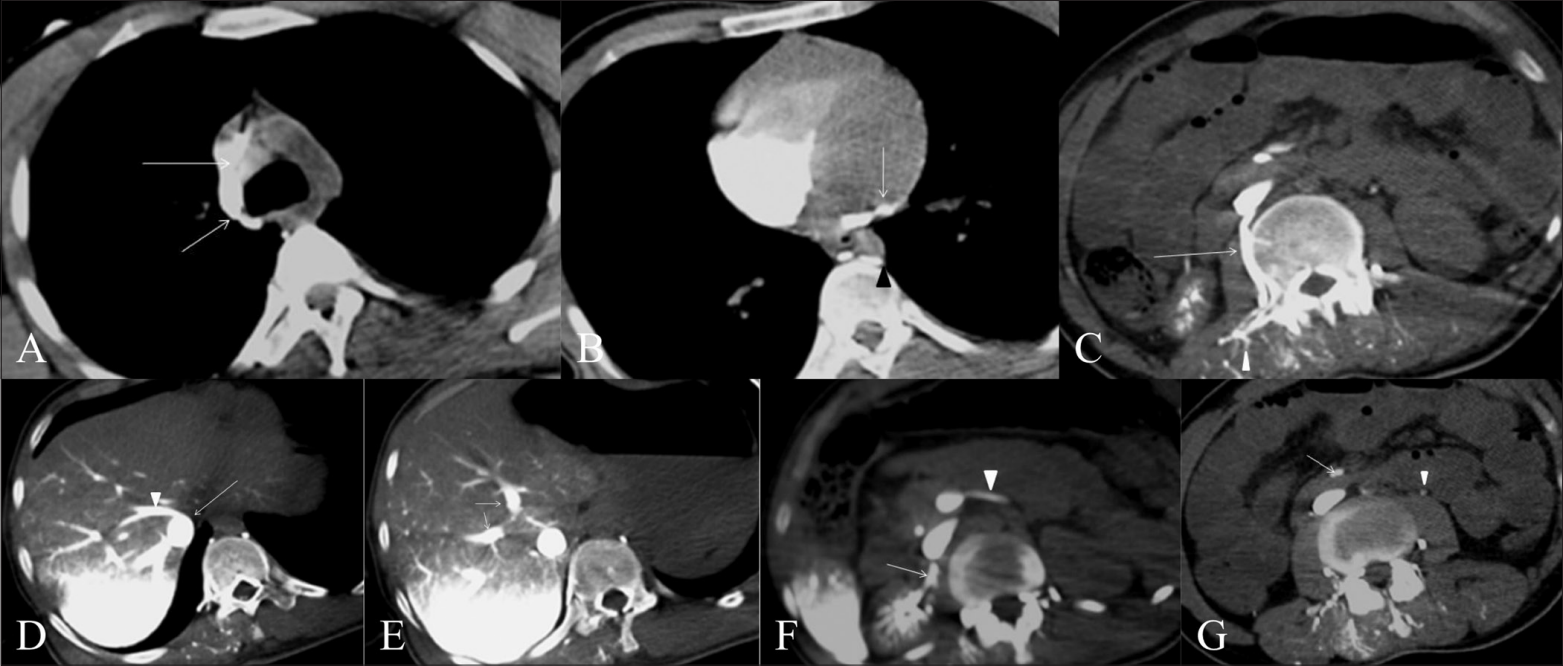
Cardiac arrest in a 26-year-old male victim of a road traffic accident. Axial CECT scans of the thorax and abdomen show dense contrast opacification of the azygos vein and SVC (arrows in A), the great cardiac (arrow) and hemiazygos (arrowhead) veins (B), the right lumbar veins (arrows in C) and the venules of the back (arrowheads in C), the IVC (arrow in D) and the hepatic veins (arrowheads in D), the portal venous branches (arrows in E), the right renal (arrow in F) and splenic (arrowhead in F) veins, and the superior mesenteric (arrow in G) and gonadal (arrowhead in G) veins. Contrast is seen to accumulate in the dependent hepatic parenchyma and in the right renal parenchyma (D–F)

The CT images revealed intense contrast opacification of the azygos and hemiazygos system, and regurgitation of contrast material from the right atrium into the great cardiac vein [Figure 2A,B]. The right chambers of the heart were opacified, but there was no contrast in the left heart chambers. Contrast was seen to accumulate in the dependent lumbar veins, the epidural veins, and dorsal veins of the back [Figure 2C]. Contrast material accumulated in the dependent portions of the SVC and the IVC, the hepatic veins, and the right renal vein; contrast also accumulated in the dependent hepatic parenchyma, with opacification of both right and left branches of the portal vein, the main portal vein, the proximal part of the splenic vein, and the superior mesenteric vein [Figure 2D–G]. The gonadal veins on both sides were also opacified [Figure 2G]. The aorta was not opacified and there was no parenchymal enhancement.

### Case 3

**Figure 3(A,B):**
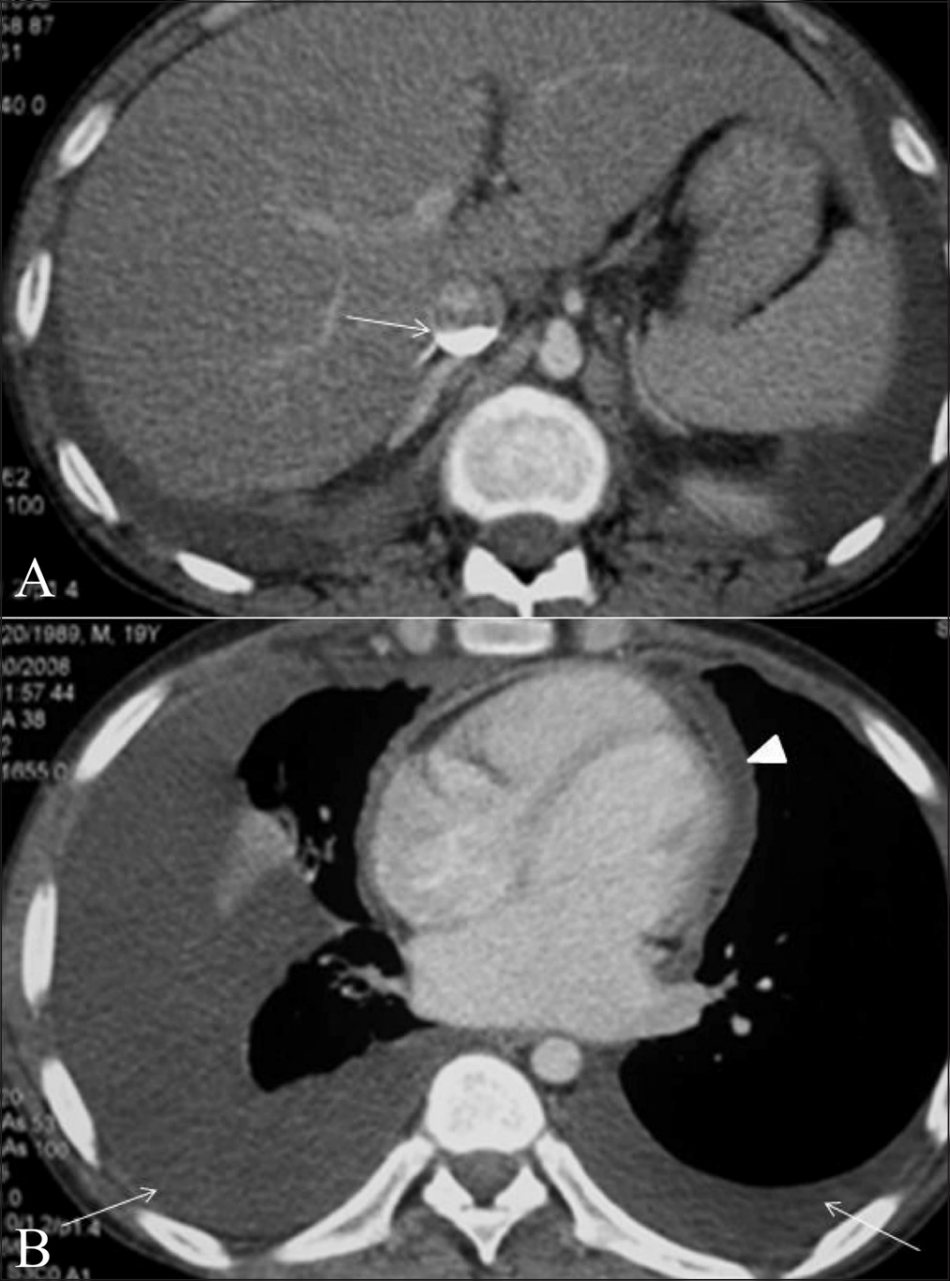
Contrast layering in the IVC (arrow in A) is seen on a contrast-enhanced, axial CT scan of a patient who died of cardiogenic shock within 24 h. He had bilateral pleural (arrows) as well as pericardial (arrowhead) effusions (B). No signs of cardiac tamponade are seen in the scan

A 19-year-old male was admitted in the medical ward with sudden worsening of chronic breathlessness. He was a known patient of rheumatic heart disease, with clinical evidence of tricuspid regurgitation. CECT scan of the chest and abdomen revealed bilateral large pleural effusions, pericardial effusion, and dependent layering of contrast material in the abdominal IVC [Figure 3A,B]. Immediate intercostal drainage of the pleural effusion was performed. The patient was sent for an emergency echocardiography, which revealed tricuspid regurgitation. He was kept under continuous monitoring of vital signs but developed cardiogenic shock and died within 24 h.

### Case 4

**Figure 4(A-C):**
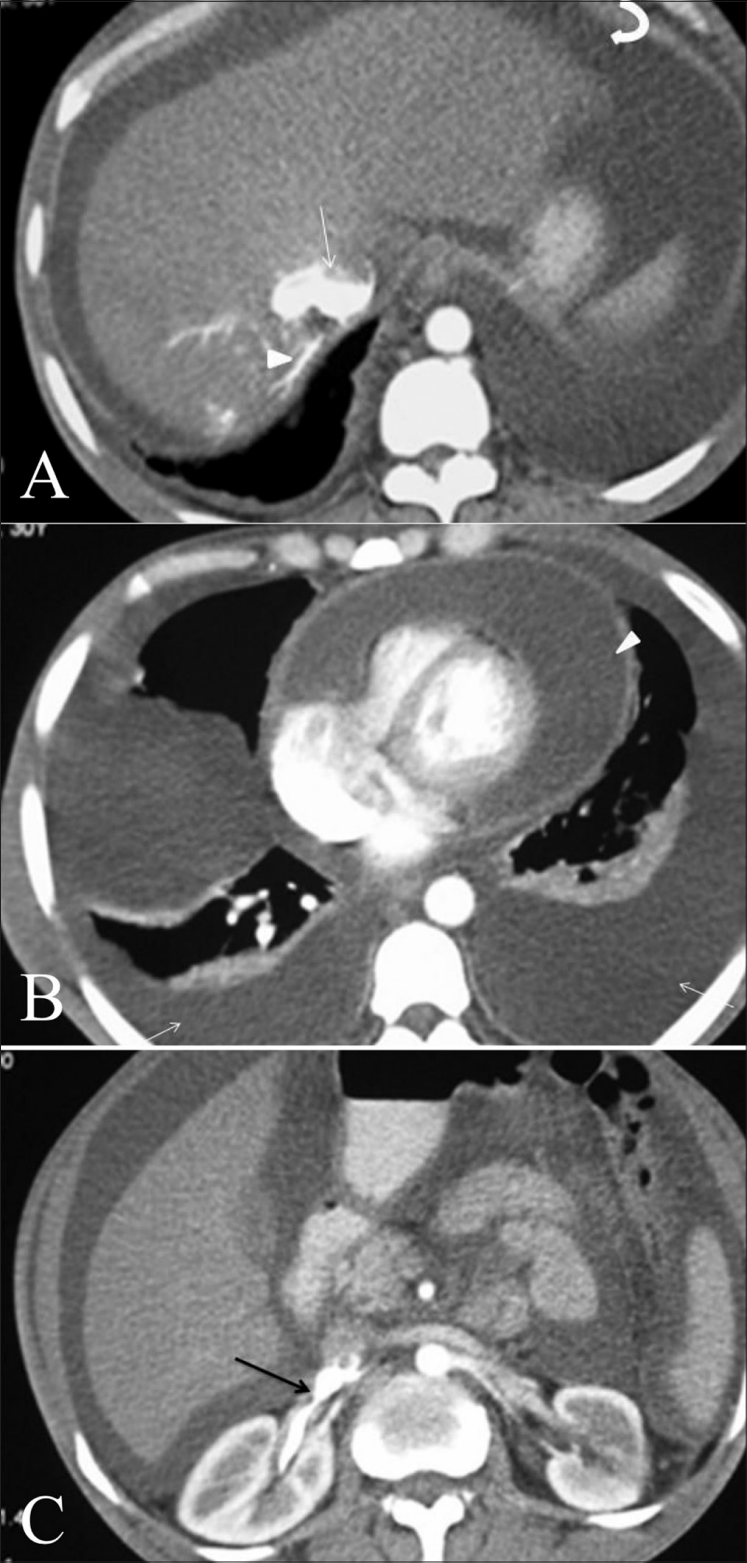
Contrast layering in the abdominal IVC (arrow) and hepatic veins (arrowhead) with ascites (curve arrow) is seen on a contrast-enhanced, axial CT scan (A), in a patient with cardiac tamponade. Gross pericardial (arrow) and bilateral pleural (arrowheads) effusions are seen (B). Contrast layering in also noted in the right renal vein (arrow in C).

A 30-year-old male, a known case of disseminated tuberculosis, presented to the emergency ward with sudden worsening of breathlessness. He was hypotensive, air entry was reduced in both lung bases, the abdomen was grossly distended, the heart sounds were muffled, the jugular veins were distended, and pulsus paradoxus was noticed, suggesting a diagnosis of cardiac tamponade. An emergency echocardiogram confirmed the presence of cardiac tamponade. A CECT scan of the thorax and abdomen revealed large bilateral pleural effusions, a large pericardial effusion, and severe ascites. Dependent contrast layering was seen in the abdominal IVC, the hepatic veins, and the right renal vein [Figure 4A,B]. The patient developed cardiogenic shock within a few hours and died despite attempts at resuscitation.

## Discussion

In cases of trauma, imaging is generally performed after primary survey and stabilization of the patient with proper resuscitation. Thus, imaging a patient during a cardiac arrest is not a common occurrence. CT scan findings of dependent venous pooling of contrast material after death or in cases of acute cardiac arrest in the CT scanner have been described previously in a few case reports in the English literature.[1–3,5] The third and fourth patients in our group were alive at the time of the study but died within 24 h. The contrast layering in IVC was a marker of imminent cardiogenic shock in this case. Similar cases have been reported previously in the literature.[4]

Cardiogenic shock is a condition of reduced tissue perfusion secondary to decreased systemic cardiac output as a result of pump failure; it occurs despite the presence of adequate intravascular volume. The causes are variable and the mortality rate is significantly high. Reflux is common in IVC and dilated hepatic veins in the setting of congestive cardiac failure and tricuspid regurgitation. Passive hepatic congestion with altered enhancement pattern has been well described in cases of right heart failure;[6,7] however, dependent venous pooling implies poor cardiac function, with failure to propel blood against gravity.[4]

When the heart stops pumping, the systemic arterial and venous pressures drop significantly, with loss of the arteriovenous pressure gradient. Hence, the distribution of injected contrast material depends on the pressure with which the contrast is injected and the density of the contrast agent.[1] Contrast material, being heavier than blood, tends to accumulate in the dependent portions of the venous system when the heart stops functioning. It has also been postulated that the positive pressure during mechanical ventilation might force the contrast material from the right heart down the IVC.[1] Contrast opacification of the portal and the superior mesenteric veins has been described previously, the most probable mechanism being retrograde filling after sinusoidal filling and opacification of the dependent parenchyma. Contrast accumulation in the splenic vein has not been described previously; in the absence of contrast in the celiac artery, the most possible mechanism is retrograde filling via the portal vein.

Although cardiogenic shock may also occur as a reaction to iodinated contrast medium administration, in all the patients who developed cardiac arrest and shock during or after CT scanning, the likely cause was unstable hemodynamics and the general physical condition, rather than an adverse reaction.

## Conclusion

Although only rarely reported, the imaging findings of sudden cardiac arrest are quite characteristic. There is little opacification of the left heart chambers and the aorta, the contrast settling down in the dependent portions of the right side of the body, predominantly in the venous system. The radiologist should be aware of the imaging findings so that proper resuscitative measures can be taken immediately in such cases, instead of continuing with the imaging. Dependent layering of contrast and the formation of a contrast-blood level carry a grave prognosis for the patient. The treating physician should be informed immediately as these findings imply imminent cardiogenic shock.
